# Impact of SARS CoV-2 /COVID-19 infection on the course of advanced chronic liver disease and hepatocellular carcinoma

**DOI:** 10.1007/s43440-022-00434-4

**Published:** 2022-11-17

**Authors:** Monika Pazgan-Simon, Marta Kucharska, Joanna Górka-Dynysiewicz, Krzysztof Simon

**Affiliations:** 1 I Department of Infectious Diseases, Jerzy Gromkowski Regional Specialist Hospital, ul Koszarowa 5, 51-149 Wrocław, Poland; 2 Department of Infectious Diseases and Hepatology, Wroclaw Medical University, Wrocław, Poland; 3 Department of Pharmaceutical Biochemistry, Wroclaw Medical University, Wrocław, Poland

**Keywords:** COVID-19, Liver cirrhosis, Hepatocellular carcinoma

## Abstract

**Background:**

About 20% of patients infected with SARS-CoV-2 develop COVID-19—the disease that has dominated health care in the last two years. The course of COVID-19 in patients with advanced liver disease tends to be severe, patients also suffer from a higher risk of complications and death. The primary object of this study was to assess the risk and causes of death in patients with cirrhosis and hepatocellular carcinoma (HCC).

**Materials and methods:**

From a group of 4,314 patients hospitalized at Jerzy Gromkowski Regional Specialist Hospital in Wroclaw (Poland) due to SARS-CoV-2/COVID-19 infection between March 15, 2020, and January 31, 2022, we selected a cohort of 31 patients with liver cirrhosis (12 women and 19 men) and 7 patients with HCC developed on the cirrhotic liver (1 woman, 6 men). The control group included 123 patients without liver disease. In the entire cohort, we analyzed the course of COVID-19 infection, baseline oxygen demand, liver function (assessed using the CTP—Child-Turoctte-Pugh score and MELD—Model of End-Stage Liver Disease scales), length of hospitalization, development of acute-on-chronic liver failure, and deaths.

**Results:**

The mean age of the patients was 56.6 years in the liver cirrhosis group, 63.3 years for patients with (HCC) hepatocellular carcinoma, and 64 years in the control group. Time of hospitalization averaged 15.52 days and 11.14 days for patients with liver cirrhosis and liver cancer, respectively. For the control group, the average duration of the hospital stay was 11.61 days. With respect to baseline liver function assessed using the CTP score, in the cirrhosis group 10 patients were CTP class A, 19 patients were class B and 9 patients were class C. The cancer group included 3 patients with class A, 2 patients with class B, and 2 patients with class C. In the studied cohort, 22 patients had a baseline MELD score < 12 points, and in 15 patients was > 12. In the HCC group, it was, respectively, CTP A:3, B: 2, C: 2, and MELD < 12: 3, ≥12: 4 people. Most of these patients presented with a progression of liver disease. Fifteen patients died, including 12 with cirrhosis and 3 with HCC, accounting for 39.47% in the entire cohort, 39% in the cirrhotic group and 43% in the HCC group, and 13 in the control group (10.6%), There was a clear statistical difference between the mortality rate in the group with liver disease and in the control group.

**Conclusions:**

Infection with SARS-CoV-2/COVID-19 in patients with cirrhosis and HCC tends to have a more severe course and leads to exacerbation of the liver disease. The most common cause of death in the analyzed cohort infected with SARS-CoV-2/COVID-19 was the progression of liver disease, complicated by liver failure.

## Introduction

SARS -CoV-2 infection and COVID-19 disease have dominated healthcare in the past two years [1]. Despite vaccine introduction, about 20% of infected patients develop pneumonia and require hospitalization. In some patients, COVID-19 is fatal, and factors that increase the risk of severe course and death are hematologic and oncologic comorbidities, heart disease, lung disease, obesity and advanced age [2].

In the course of COVID-19, many patients (20–70%) develop liver damage which is most often manifested by an increase in aminotransferase activity, GGTP and bilirubin levels [3, 4]. The possible reasons for hepatic pathologies in this group of patients are cytotoxic damage to liver cells, hypoxia of these cells, and formation of clots in the portal spaces [5, 6], Similarly, many medications used in COVID-19 therapy–such as remdesivir, tocilizumab, azithromycin or corticosteroids–have a liver-damaging effect [7–9]. Hepatopathy can also be an element of cytokine storm and multi-organ failure. In most patients, liver damage is transient and usually does not increase mortality, except for the cases with cytokine storm and in the sporadic cases of drug-induced hepatopathies.

Patients with liver cirrhosis and hepatocellular carcinoma (HCC) are rather complex groups, burdened with a significant impairment of multiple liver functions and dysfunction of other organs, as well as with the cirrhosis-associated immune dysfunction syndrome (CAIDS), which may lead to a more severe course of COVID-19 and increase the risk of death. Patients who require hospitalization and a subsequent Intensive Care Unit (ICU) stay are particularly at risk. In some patients, complications concern the respiratory system, but the majority suffer from acute-on-chronic liver failure followed by death [10]. In patients with liver cancer, SARS-CoV-2 infection also leads to delays and interruptions in oncological therapy, adversely affecting the entire course of the primary disease [11].

In our study, we analyzed the course of COVID-19 in patients with advanced liver disease (in this paper meaning both advanced liver disease and liver cirrhosis) and HCC. All patients were citizens of Poland, Caucasian, and required hospitalization due to COVID-19 infection.

The primary object of this study was to assess the risk and causes of death in patients with cirrhosis and HCC (research cohorts) as compared with the course of COVID-19 in patients with no history of liver disease or other significant diseases.

Additional objectives of the analysis were a 3-point assessment of the degree of liver function on CTP and MELD scores: baseline, after 7 and after 14 days of hospitalization for COVID-19, and to monitor and compare the duration of hospitalization in the cohort and in the control group.

## Materials and methods

The research consisted of 31 patients with liver cirrhosis (12 women; 19 men)–sample I–and 7 patients (1 woman and 6 men) with liver cirrhosis complicated by hepatocellular carcinoma–sample II–selected from the group of 4314 COVID-19 patients hospitalized between March 15, 2020, and January 31, 2022, in Jerzy Gromkowski Regional Specialist Hospital in Wroclaw, the main center for COVID-19 in the Lower Silesia region in Poland. Due to the increasing variability of SARS-CoV-2 in the course of the pandemic, i.e., different infectivity and pathogenicity of subsequent virus variants, the control group comprised 123 patients (the first 25 patients from each half of 2020 and 2021 and 23 patients from the first half of 2022) hospitalized at Jerzy Gromkowski Regional Specialist Hospital in Wroclaw for SARS-CoV- 2/COVID-19 infection, admitted without baseline liver disease and other significant comorbidities (except hypertension).

Ethics committee approval was received for this study from the Bioethics Committee at the Lower Silesian Medical Chamber in Wroclaw (ID KB / 242/2020). Patients consented to participate in the study.

The diagnosis of liver cirrhosis and hepatocellular carcinoma (HCC) was made prior to the hospitalization, on the basis of generally accepted diagnostic principles. The SARS-CoV-2 infection was confirmed by a PCR test in all patients—who required hospitalization due to clinical and radiological symptoms of pneumonia and a decrease in Sat O2 < 94%.

We analyzed the following endpoints in the entire cohort: course of COVID-19 infection, baseline oxygen demand (measured with a pulse oximeter), liver function calculated using CTP (Child-Turcotte-Pugh) score (bilirubin and albumin concentrations, encephalopathies, ascites) and MELD score (creatinine and bilirubin concentrations, INR), duration of hospitalization, development of acute-on-chronic liver disease (liver decompensation in the form of: encephalopathy, ascites, gastrointestinal bleeding, acute kidney injury–AKI) and causes of death. The analysis involved account vaccinations against COVID, although a subgroup of patients was hospitalized before the vaccines became available. We also described the complications of oncological treatment that the patients with HCC experienced during COVID-19.

The results obtained were statistically analyzed using Statistica 13.3 software (StatSoft, Kraków, Poland). The Shapiro–Wilk normality test was used to check the normality of data distributions. Since not all measured data were normally distributed, nonparametric tests were used. The chi-square test was used for the comparison of differences. The Fisher Exact Probability Test was used to verify if proportions of one variable are significantly different depending on the value of the other variable. The value of *p* < 0.05 was considered statistically significant.

## Results

Among a large number (over 4000) of patients with Covid-19 hospitalized at Jerzy Gromkowski Regional Specialist Hospital, there were only 38 persons (25 men and 13 women) with advanced liver disease. Sample I—31 people: (19 men and 12 women) were patients with liver cirrhosis only, sample II—7 people (6 men and 1 woman): were patients with hepatocellular carcinoma developed on liver cirrhosis and the control group comprised 123 people infected with COVID-19 (67 men and 56 women). The number of patients vaccinated against SARS-CoV-2 (with a different number of doses, ranging from 1 to 4) was as follows: sample I—15 out of 31, sample II—5 out of 7, control group—48% ( 59 out of 123). Baseline liver function was assessed using two scoring models. In sample I (liver cirrhosis) it was as follows—CTP score: Class A (3–6 points)—7 patients, Class B (7–9 points)—17 patients, and Class C—7 (10 points and above) – patients, MELD score: < 12 point—20 patients, equal or > 12–11 patients. In sample II (HCC) it was as follows, respectively, CTP score: Class A—3, Class B—2, Class C—2 patients, MELD score: < 12 points—3, equal or > 12 points—4 patients. Data are presented in Table 1.

**Table 1:** Characteristics of the research cohort* (sample I— cirrhotic patients and sample II—patient with hepatocellular carcinoma (HCC)): gender, vaccination against Covid -19 and death

	Female/Male, % (*n*)	Vaccination against COVID-19, % (*n*) Y/N	Death, % (*n*) Y/N	CTP, % (*n*)		MELD, % (*n*) < 12/ > 12
All patients *n* = 38	34.2 (13)/65.8 (25)	44.7 (17)/55.3 (21)	39.5 (15)/60.5 (23)	A	26.3 (10)	60.5 (23)/39.5 (15)
				B	50.0 (19)	
				C	23.7 (9)	
Liver cirrhosis (I) *n* = 31	38.7 (12) /61.3 (19)	48.4 (15)/ 51.6 (16)	38.7 (12)/61.3 (19)	A	22.6 (7)	64.5 (20)/35.5 (11)
				B	54.8 (17)	
				C	22.6 (7)	
HCC (II) *n* = 7	14.3 (1) /85.7 (6)	71.4 (5)/28.6 (2)	42.9 (3)/57.1 (4)	A	42.8 (3)	42.9 (3)/57.1 (4)
				B	28.6 (2)	
				C	28.6 (2)	

(CTP) Child-Turcotte**-**Pugh score, (MELD) Model of End-Stage Liver Disease scales. CTP: class A (well-compensated disease), class B (significant functional compromise), class C (decompensated disease)

*Patients hospitalized between March 15, 2020, and January 31, 2022, in Jerzy Gromkowski Regional Specialist Hospital in Wrocław (Poland)

The mean age in patients with the advanced liver disease was 56.64 years (35–75 years) and in patients with hepatocellular carcinoma it was 63.3 years (59–71 years). The mean age in the control group was 64.19 years (27–97). Time of hospitalization averaged 15.52 days (2–58 days) and 11.14 days (4–21 days) for patients with liver cirrhosis and liver cancer, respectively. For the control group, the average duration of the hospital stay was 11.61 days (1–60). Duration of hospitalization was statistically longer in the study group (cirrhosis and HCC) compared to the control group (*p* = 0.03265). The etiology of chronic liver disease was diverse, including alcoholic liver disease as the main cause in 19 out of 38 patients, followed by non-alcoholic fatty liver disease (5 cases) and HBV infection (4 cases) HCV infection, and mixed etiology were less common. Comorbidities are presented in Table 2.

**Table 2:** Epidemiological and demographic characteristics of the entire analyzed cohort*

Parameters	Control group—patients without liver disease *n* = 123	Liver cirrhosis (I) *n* = 31	HCC (II) *n* = 7
Age (years)	64.19 (27–97)	56.64 (35–75)	63.29 (59–71)
Duration of hospitalization (days)	11.61 (1–60)	15.52 (2–58)	11.14 (4–21)
Etiology	–	HBV–4 HCV–2 HBV/HCV–1 NAFLD–3 ALD–20 AIH–5 HBV/AIH–2 HCV/NAFLD–1	
Comorbidities	*n* (%)	*n* (%)	
Hypertension	53 (43.09%)	12 (31.56%)	
Heart disease	6 (4.88%)	6 (15.79%)	
Diabetes mellitus	15 (12.19%)	22 (57.89%)	
Complications of diabetes	–	2 (5.26%)	
Ulcerative colitis	–	1 (2.63%)	
Metabolic syndrome	–	4 (2.63%)	
Cholecystolithiasis	–	2 (5.26%)	
Atherosclerosis	–	4 (10.53%)	
Epilepsy	1 (0.81%)	3 (7.89%)	
Hashimoto’s disease	3 (2.44%)	2 (5.26%)	
Hypothyroidism	3 (2.44%)	1 (2.63%)	
Pancreatitis	–	5 (13.16%)	
Anemia	–	1 (2.63%)	
HIV	–	1 (2.63%)	
Discopathy	–	1 (2.63%)	
Prostatic hyperplasia	2 (1.63%)	1 (2.63%)	
Central pontine myelinolysis	2 (1.63%)	2 (5.26%)	
Asthma	6 (4.88%)	1 (2.63%)	

Continuous variables are expressed as median, range; categorical variables as number (percentage)

*Patients hospitalized between March 15, 2020, and January 31, 2022, in Jerzy Gromkowski Regional Specialist Hospital in Wrocław (Poland)

Upon admission, 36 out of 38 patients required oxygen therapy. Out of these 36 patients, one patient required high-flow oxygen therapy. During hospitalization, two male patients developed respiratory failure, required intubation, and were transferred to the ICU (both cases ended in deaths). In the control group, 9 patients required high-flow oxygen therapy, and 3 required intubation.

All patients received inhaled and intravenous glucocorticosteroids, low-molecular-weight heparin, low-dose statins, and oral or intravenous paracetamol. Only two patients in sample A received antiviral medication. The other patients manifested the symptoms long before admission, therefore, that was no justification for antiviral treatment. Additionally, antiviral therapies have their limitations. For example, remdesivir is contraindicated in patients with significantly elevated aminotransferase levels, malnupiravir is contraindicated in thrombosis (vessel blood clots in portal vein confluence are commonly found in patients with cirrhosis and HCC). In contrast, as many as 25 out of 123 (20%) patients in the control group met the safety criteria and received remdesivir treatment. All patients with signs of cytokine storm (2 patients in sample I, and 3 patients in the control group) were treated with tocilizumab, provided that they met the safety criteria for such therapy.

Sixteen patients in the cirrhosis sample and two in the liver cancer group experienced clinical progression of liver disease which we also assessed using CTP and MELD scores. The progression included the development of acute-on-chronic liver disease. Namely, ascites occurred in 5 patients (one with HCC), encephalopathy occurred in 5 patients (one with HCC), portal thrombosis occurred in one patient, and gastrointestinal bleeding occurred in 3 patients. Six patients developed acute renal failure (2 patients with HCC) and 4 developed sepsis, including septic shock in one case. Of the non-hepatic complications, one patient developed a *Clostridioides difficile* infection and an episode of atrial fibrillation was observed in one case.

In the HCC—sample II, 3 out of 7 patients were undergoing oncological treatment prior to SARS-CoV-2 infection, in the remaining patients the disease was either too advanced for therapy or they gave up treatment on their own (two patients suffered from alcohol addiction). In two patients, the SARS-CoV-2/COVID-19 infection resulted in a delay (longer than a month) in treatment, medical imaging, and oncological therapy. One patient was scheduled for HCC resection, but due to the delay caused by COVID-19 and the progression of liver disease, was disqualified from surgery and started chemotherapy. During the COVID-19 infection, systemic HCC treatment was discontinued in the remaining two patients on chemotherapy. Both patients suffered delays in scheduled x-ray imaging, and their cancer processes progressed due to the suspension of therapy. However, later on, both patients resumed treatment and their status is stable. Detailed data on HCC patients are presented in Table 3.

**Table 3:** Characteristics of hepatocellular carcinoma (HCC) patients (sample II) *

Gender (M-male, F-female)	Viral etiology	HBV/HCV treatment	Alcohol intake	Child-Turcotte-Pugh score	MELD score	Portal vein thrombosis	BCLC	AFP (ng/ml)	Nourishment level	HCC treatment
M	HCV	Resolved	No	A, 5	9	No	B	7	Normal	Sorafenib
M	No	NA	No	A, 5	9	No	C	1313	Protein-calorie malnutrition	PD-1 blocker, Apatinib
M	No	NA	Yes	C, 10	15	No	C	3031	Protein-calorie malnutrition	None
F	No	NA	No	B, 8	23	Yes	C	13,200	Protein-calorie malnutrition	None
M	HCV/HBV	Active, not treated HCV infection	Yes	B, 7	14	Yes	C	48	Emaciation	None
M	HBV	Entekavir	No	A, 5	12	No	C	28	Normal	Plan to surgery
M	No	NA	Yes	B, 8	11	No	C	6500	Protein-calorie malnutrition	None

A total Child-Turcotte-Pugh score of 5 to 6 is considered Child–Pugh class A (well-compensated disease), 7 to 9 is class B (significant functional compromise), and 10 to 15 is class C (decompensated disease).MELD score (- consider liver transplant if more than 12 points). BCLC 0—very early stage, A—early stage, B- intermediate- stage, C- advanced stage, D terminal stage

*Patients hospitalized between March 15, 2020, and January 31, 2022, in Jerzy Gromkowski Regional Specialist Hospital in Wrocław (Poland)

In the liver cirrhosis sample, two patients develop cytokine storm (progression of infectious inflammation, IL-6 increase: 480 pg/ml in the first patient and 280 pg/ml in the second patient). The patients were transferred to ICU, and received tocilizumab treatment, yet died due to liver failure. In the control group cytokine storm was observed in three patients (IL-6, respectively: 123 pg/ml, 217 pg/ml, and 134 pg/ml) who then received tocilizumab, one patient required intubation and stayed at the ICU, all three survived.

The deaths during hospitalization included twelve patients (39.47%) in the liver cirrhosis sample (out of which half were female) and three patients with liver cancer (one female), in the total analyzed cohort 53.85% of females, and 32% of males died. In the control group, the proportionate mortality was 10.6% (10.7% females; 9.0% males), and the differences were statistically significant (Fisher Exact Probability Test, females *p* = 0.002, males *p* = 0.018, total *p* < 0.001). In sample I the proportionate mortality was 39.47% (53.85% females; 32.0% males) and the differences were statistically significant (Fisher Exact Probability Test, females *p* = 0.005, males *p* = 0.021, total *p* < 0.001). Due to the small sample size, we did not calculate statistical significance for HCC (sample II) patients. Detailed data are presented in Table 4.

**Table 4:** Mortality in patients with liver cirrhosis (I) and hepatocellular carcinoma (II) compared to the control group*

	Control group – patients without liver disease			Total analyzed cohort (liver cirrhosis/ HCC)				Liver cirrhosis				HCC		
	Sample size *n*	Deaths *n*	Proportion of deaths (%)	Sample size *n*	Deaths *n*	Proportion of deaths (%)	**p-value	Sample size *n*	Deaths *n*	Proportion of deaths (%)	**p-value	Sample size *n*	Deaths *n*	Proportion of deaths (%)
Total	123	13	10.6%	38	15	39.47%	0.000	31	12	38.71%	0.001	7	3	42.86%
Women	56	6	10.7%	13	7	53.85%	0.002	12	6	50.00%	0.005	1	1	100.00%
Men	67	6	9.0%	25	8	32.00%	0.018	19	6	31.58%	0.021	6	2	33.33%

A total Child-Turcotte-Pugh score of 5 to 6 is considered Child–Pugh class A (well-compensated disease), 7 to 9 is class B (significant functional compromise), and 10 to 15 is class C (decompensated disease)

*Patients hospitalized between March 15, 2020, and January 31, 2022, in Jerzy Gromkowski Regional Specialist Hospital in Wrocław (Poland)

**Fisher Exact Probability Test

Deaths in the research samples (calculated as a whole) were related to patients’ age (there were no deaths in patients < 45 years). In the liver cirrhosis sample, the number of deaths showed a statistically significant relationship (Chi-square test of independence, advanced liver disease assessed using CTP and MELD and in the HCC sample, CTP *p* = 0.02, and MELD—*p* = 0.014). No differences were observed between patients vaccinated/not-vaccinated against COVID-19 (Chi-square test of independence *p* = 0.7), and also when the cohort was broken down by gender (*p* = 0.52).

## Discussion

A total of 2% of the patients hospitalized due to SARS-CoV-2/COVID-19 infection at the Jerzy Gromkowski Regional Specialist Hospital (Wrocław, Poland) were patients with liver cirrhosis and liver cirrhosis complicated with HCC. This percentage is 20 times higher than the estimated percentage of cirrhotic patients observed in the Polish population (540,000 patients in a population of 38 million) and 15 times higher than the percentage of HCC patients in Poland (8 out of 1,463 patients) [12]. These data indicate that patients with liver cirrhosis and HCC are at a higher risk of experiencing a severe course of SARS-CoV-2 infection; the demand for hospitalization due to COVID-19 is also greater in this group.

We observed that SARS-CoV-2/COVID-19 infection is associated with high mortality in both patients with liver cirrhosis and hepatocellular carcinoma (HCC). Observations on the course of COVID-19 in cirrhotic, patients in Poland are scarce and no data are available on the course of COVID-19 in patients with liver cirrhosis complicated with HCC. Nevertheless, our observations are confirmed by results published by other authors, who show that liver cirrhosis and HCC are independent factors increasing the severity of the course of COVID-19, which is associated with a higher risk of hospitalization and higher mortality. HCC alone, regardless of the coexistence of liver cirrhosis, is also associated with a higher risk of death in COVID-19 patients [13]. Patients with HCC, regardless of stage, are also at a higher risk of infection with SARS-CoV-2 than the general population [14]. Surprisingly, most of the patients in the analyzed samples, both with cirrhosis and HCC, required passive oxygen therapy only; only one patient needed high-flow oxygen therapy, and two patients developed a lung disease progression that resulted in intubation and ICU treatment. In most cases, deaths in the course of SARS-CoV-2/COVID-19 in this group of patients were not resulting from respiratory failure followed by ARDS—which is typical for severe forms of SARS-CoV-2 infection. These deaths were caused by the progression of liver disease in the acute-on-chronic liver failure mechanism (ACLF) and, ultimately, its failure—especially in elderly patients. ACLF is an inflammatory disorder induced by various factors: viral and bacterial infections, chemical agents including drugs, induced aggressive autoimmune phenomena—and thus a complex pathomechanism. It occurs suddenly in patients with underlying liver disease and is characterized by frequent failure of the liver and other organs. It has been shown that 12–50% of patients with COVID-19 and chronic liver disease develop ACLF, and the severity of liver disease was also a strong predictor of the severe course of COVID-19 [15]. If we look at our research cohort, most patients seem to meet the criteria to qualify for this mechanism. Similarly, higher CTP and MELD scores, indicating more advanced liver pathologies, were associated with a higher risk of death [23]. In a group of 103 patients with liver cirrhosis and COVID-19, Moon et al. [23] observed a significant increase in the risk of death, following an increase in the CTP score. Proportionate mortality was as follows: in patients with CTP class A—24%, CTP class B—numery43%, and CTP class C—as much as 63%. However, contrary to our observations, Moon et al. [23] concluded that only 12.2% of patients died directly from the progression of liver disease and liver failure and that 78.7% of deaths were due to lung disease and 4.3% died due to cardiovascular issues. In another multi-center study, Bajaja et al. [16], analyzed the risk of death in patients divided into three samples: patients with cirrhosis and COVID-19 (37 people), patients with isolated cirrhosis and without concomitant infection (127 people) and patients with isolated COVID-19 infection (108 people). The authors showed that the risk of death in patients with cirrhosis was similar, regardless of additional factors such as COVID-19 infection, but higher than in patients infected with COVID-19 and without accompanying advanced liver disease. These observations are consistent with our results and indicate that liver cirrhosis is an independent predictor of death in the course of COVID-19, and that high mortality in this group of patients is mainly related to the advanced stage of liver disease and its progression.

On the other hand, it is worth taking a closer look at patients with liver cirrhosis who developed a cytokine storm. In our study, all patients with cirrhosis who experienced it, required an ICU transfer and died. The possible reasons include an exacerbation of baseline immune system disorder.

The effect of a cytokine storm on mortality in patients with liver cirrhosis and hepatic insufficiency is difficult to investigate and appears in few scientific publications. Cytokine storms are also associated with a high risk of thromboembolic complications [17].

Hemostatic disorders are observed at the very early stages of liver cirrhosis [18]. They are caused by impairment of the synthetic function of the liver and complications of cirrhosis that take the form of portal hypertension and abnormal endothelial function. Since synthesis disorders concern both the pro-thrombotic and the anti-thrombotic factors, their deficiencies balance out, which means that a state of relative equilibrium can be observed. However, under ischemic and hypoxic conditions, hepatocytes fail to produce normal amounts of the major physiological anticoagulant, protein C and its cofactor, protein S. Additionally, damage to endothelial cells in portal hypertension cause activation and consumption of these proteins. Increased levels of inflammatory mediators result in vasculitis and flow deceleration in the veins and the portal system, leading to stasis [18]. Additionally, in the course of hepatic failure, D-dimer and fibrin degradation product values are increased [19].

These abnormalities make the group of patients with cirrhosis and its failure particularly sensitive to thromboembolic and hemorrhagic complications, which is confirmed in research published on the topic. It has been widely shown that patients with liver cirrhosis are predisposed to microthrombosis of the lungs and thromboembolic events [20]. Due to hyperdynamic circulation and endothelial inflammation, patients with decompensated cirrhosis are also at increased risk of thromboembolic complications [21]. The influx of inflammatory mediators alone increases the risk of deep vein thrombosis in cirrhosis and liver failure.

At the same time, endogenous heparinoids are produced in patients with hepatic failure followed by cytokine storm or with cirrhosis followed by systemic inflammation, which affects coagulation and predisposes to bleeding [22]. It is consistent with our observations It has been proved that—in addition to progressive respiratory failure and secondary multi-organ failure or sepsis—bleeding from esophageal varices is among the leading causes of death in patients with cirrhosis and COVID-19 [23].

It is also worth paying attention to another additional element of therapy. In the control group, twenty patients received remdesivir treatment and one died (1/20), while in the group with cirrhosis two patients were treated with remdesivir and one died. Despite the undoubtedly limited effectiveness of remdesivir in preventing the progression of COVID, the majority of patients with advanced liver disease could not receive antiviral therapy [24].

Yet another significant issue is how SARS-CoV-2/COVID-19 infection influences the ongoing oncological therapy in HCC patients. Looking at the problem from a global perspective, 38% of oncological procedures were not performed or were postponed due to COVID-19 [11]. In a systematic review, based on a number of studies, Riera et al. showed that 77.5% of HCC cancer patients suffered at any stage of the diagnosis and treatment. This was confirmed by our observations. Alterations to HCC therapy were experienced by all patients in our HCC sample who survived COVID-19 [25]. For one patient, a resection procedure was delayed. Since the SARS-CoV-2 viral load in his case was longer than four weeks, HCC rapidly progressed and the patient was eventually disqualified from surgery altogether. For the remaining three patients infected with COVID-19 cancer therapy was temporarily suspended; they also failed to undergo necessary x-ray imaging.

A limitation of this research is the unexpectedly small size of research samples and the presence of different various virus variants during the observed hospitalization period, which hindered the process of conclusion- making.

## Data Availability

Data are available on request from the corresponding author. The data are not publicly available due to privacy or other restrictions.

## Abbreviations

AIH: Autoimmune hepatitis
ALD: Alcoholic liver disease
HBV: Hepatitis B virus
HCC: Hepatocellular carcinoma
HCV: Hepatitis C virus
CTP: Child-Turcotte-Pugh score
ICU: Intensive Care Unit, Il-6 interleukin 6
NAFLD: Non-alcoholic fatty liver disease
MELD: Model For End-Stage Liver Disease score
